# Robotic Pancreaticoduodenectomy Using the *hinotori* Surgical System with Extended Resection for Type II Circumportal Pancreas: A Case Report

**DOI:** 10.70352/scrj.cr.25-0663

**Published:** 2025-12-23

**Authors:** Noriyuki Egawa, Takao Ide, Tomokazu Tanaka, Sachiko Nishinaka, Keita Kai, Hirokazu Noshiro

**Affiliations:** 1 Department of Surgery, Faculty of Medicine, Saga University, Saga, Saga, Japan; 2 Department of Pathology & Microbiology, Faculty of Medicine, Saga University, Saga, Saga, Japan; 3 Department of Pathology, Saga University Hospital, Saga, Saga, Japan

**Keywords:** robotic pancreaticoduodenectomy, circumportal pancreas, minimal invasive surgery

## Abstract

**INTRODUCTION:**

Circumportal pancreas (CP) is a rare congenital anomaly in which pancreatic parenchyma encases the portal vein. CP poses significant technical challenges during pancreatic surgery, particularly in safely isolating the pancreas from the portal vein, determining the optimal transection line, and managing the pancreatic stump. This report describes a case of CP identified intraoperatively during a robotic pancreaticoduodenectomy (RPD), in which an extended resection was required to achieve a single pancreatic duct for safe reconstruction.

**CASE PRESENTATION:**

A 77-year-old man diagnosed with distal bile duct cancer underwent RPD using the *hinotori* Surgical Robot System (Medicaroid, Hyogo, Japan). Preoperative contrast-enhanced CT demonstrated circumferential encasement of the portal vein by pancreatic parenchyma, findings that could have suggested the presence of a CP. However, these findings were not recognized at the preoperative stage, and the diagnosis of CP had not been established before surgery. During surgery, when the pancreas was found to encircle the portal vein, retrospective review of the imaging confirmed the presence of CP. However, the courses of the main and accessory pancreatic ducts remained indeterminate on both imaging and intraoperative findings, making classification according to Karasaki’s system difficult. After careful dissection and mobilization of the pancreatic body from the portal vein, an extended resection was performed to obtain a single pancreatic duct for reconstruction. Pancreaticojejunostomy was performed using a standard duct-to-mucosa anastomosis with the modified Blumgart technique for a single pancreatic duct. The operation time was 1050 minutes, and the estimated blood loss was 795 mL. Histopathological examination revealed distal bile duct adenocarcinoma (pStage IIA, T2N0M0) with type II CP, in which the main pancreatic duct traversed the dorsal pancreas and the accessory duct ran ventrally. The postoperative course was uneventful, and the patient remains disease-free 18 months after surgery.

**CONCLUSIONS:**

When the MPD course is unclear in CP, extended resection represents a rational and safe surgical strategy to achieve single-duct reconstruction. Although such complex cases have traditionally been managed by open surgery, robotic surgery enables safe and minimally invasive pancreaticoduodenectomy even in anatomically challenging conditions. To our knowledge, this is the 1st reported case of robotic pancreaticoduodenectomy for type II CP.

## INTRODUCTION

Circumportal pancreas (CP) is a rare congenital anatomical variation^1–3)^ that complicates pancreatic resection, particularly in safely isolating the pancreas from the portal vein, determining the optimal transection site, and managing the transected stump^3–5)^ (**Supplementary Video 1**). Preoperative imaging plays a crucial role in identifying this anomaly, and a thorough understanding of the pancreatic duct course is essential for safely determining the transection plane. However, accurate preoperative diagnosis remains challenging, and CP is often recognized only intraoperatively.^4,5)^ Failure to appropriately address this variation can result in significant technical difficulties and postoperative complications.

In this report, we present a case of CP that was identified intraoperatively during robotic pancreaticoduodenectomy (RPD) for distal bile duct cancer. The absence of main pancreatic duct (MPD) dilation made it difficult to delineate its course within the pancreatic head on imaging, necessitating an extended resection.^3,5–8)^ Pathological examination confirmed type II CP.^2)^ The optimal pancreatic transection plane during pancreaticoduodenectomy for CP has been discussed based on the MPD course,^3–10)^ however, as demonstrated in this case, the MPD course may remain indeterminate despite thorough preoperative imaging and careful intraoperative assessment.

This case provides valuable insights into determining the optimal pancreatic transection plane during pancreaticoduodenectomy in patients with CP when the MPD course cannot be identified preoperatively. Furthermore, to the best of our knowledge, this is the 1st reported case of RPD for type II CP, and we describe the detailed surgical techniques applied.

## CASE PRESENTATION

### Case

The patient was a 77-year-old male who presented to another hospital with fever and jaundice. He was diagnosed with obstructive jaundice and cholangitis caused by distal bile duct cancer. Endoscopic retrograde biliary drainage (ERBD) was performed for biliary decompression. After the resolution of cholangitis, he was referred to our hospital for surgical intervention.

**Fig. 1:**
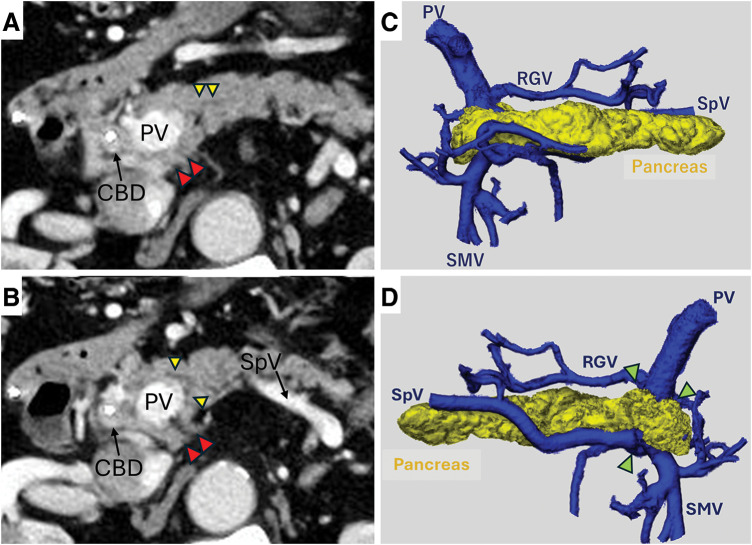
Contrast-enhanced CT and 3D CT findings of CP. (**A**) Thickened CBD wall with a biliary stent. Aberrant pancreatic parenchyma extending posterior to the PV corresponds to the characteristic findings of CP (red triangles). (**B**) No dilation of the MPD (yellow triangles) is detected, and its course within the pancreatic head remains indeterminate, making Joseph's classification inconclusive. (**C**) Anterior view of 3D CT shows pancreatic parenchyma encircling the PV. (**D**) Posterior view reveals the fusion plane cranial to the SpV (green triangles), consistent with Kawasaki’s type A classification. CBD, common bile duct; CP, circumportal pancreas; MPD, main pancreatic duct; PV, portal vein; RGV, right gastric vein; SMV, superior mesenteric vein; SpV, splenic vein

Abdominal CT revealed a 2.2 × 1.5 cm mass in the distal bile duct, accompanied by bile duct dilation. There was no dilation of the MPD, and its course within the pancreatic head could not be identified (**Fig. 1B**). Additionally, CT demonstrated pancreatic parenchyma encircling the portal vein, consistent with CP (**Fig. 1A**, **1C**, and **1D**; **Supplementary Video 2**); however, this finding was not recognized preoperatively.

The patient underwent RPD using the *hinotori* Surgical Robot System (Medicaroid, Hyogo, Japan). The operation time was 1050 minutes, and the estimated blood loss was 795 mL. The prolonged operative time was mainly due to dense adhesions around the bile duct caused by preoperative cholangitis.

**Fig. 2:**
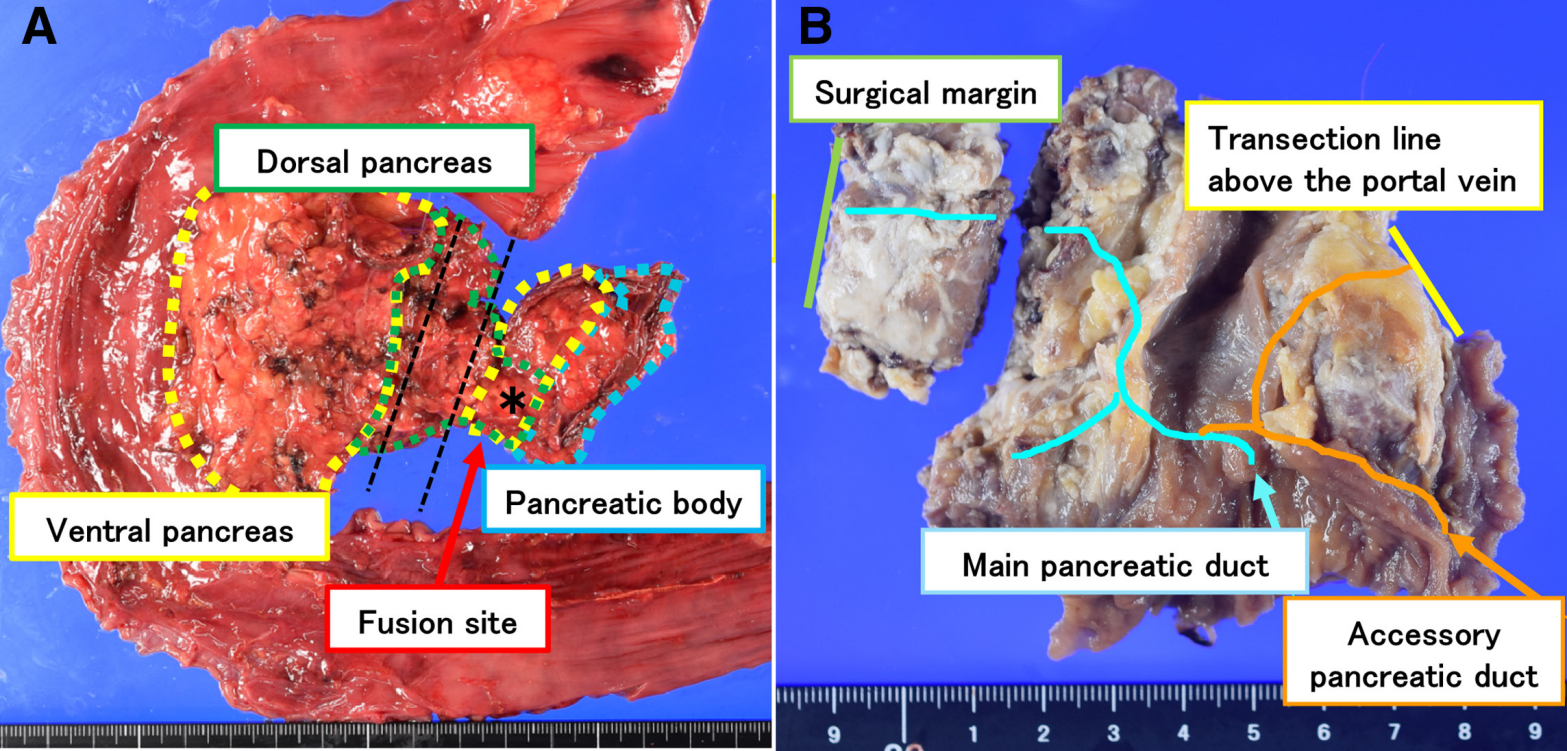
Macroscopic pathological findings of the resected specimen. (**A**) The PV and SMV course within the black dotted line, and the asterisk (*) indicates the fusion plane between the ventral and dorsal pancreas. (**B**) The MPD (blue line) traverses the dorsal pancreas, while the APD (orange line) runs through the ventral pancreas and opens into the minor duodenal papilla. The yellow line denotes the initial pancreas transection plane, positioned just above the PV/SMV, and the red line marks the distal resection margin. APD, accessory pancreatic duct; MPD, main pancreatic duct; PV, portal vein; SMV, superior mesenteric vein

Histopathological examination revealed distal bile duct adenocarcinoma (pStage IIA (T2N0M0)) with negative surgical margins. The specimen also showed that the MPD traversed the dorsal pancreas, while the accessory pancreatic duct, running through the ventral pancreas, opened into the minor duodenal papilla (**Fig. 2A** and **2B**). These findings led to the diagnosis of type II CP according to Joseph’s classification.^2)^

Postoperative recovery was uneventful, with no pancreatic fistula, and the patient was discharged on POD 22. He has since received adjuvant chemotherapy and remains disease-free 18 months after surgery.

### Surgical technique

**Fig. 3:**
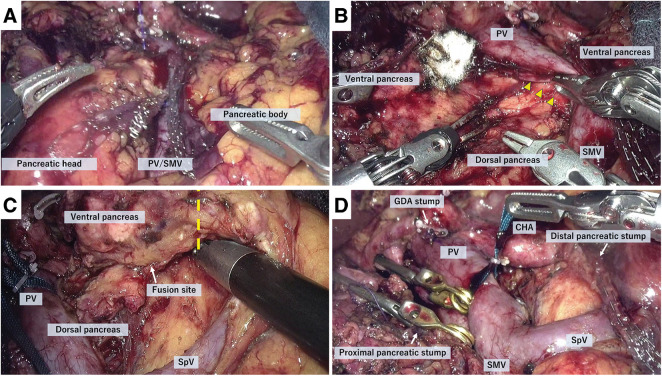
Intraoperative findings of RPD with extended pancreas resection. (**A**) The initial transection of the pancreatic parenchyma was performed above the PV, however, the presence of CP was not identified at this stage. (**B**) The pancreatic parenchyma of the uncinate process extended continuously from the posterior aspect to the left side of the PV, fusing with the pancreatic body (yellow triangles). At this point, CP was identified. (**C**) “Extended resection” was performed beyond the dorsal–ventral fusion plane, with the yellow dotted line indicating the transection plane. (**D**) Final view after completing RPD with extended pancreas resection. CHA, common hepatic artery; CHD, common hepatic duct; CP, circumportal pancreas; GDA, gastroduodenal artery; PV, portal vein; RPD, robotic pancreaticoduodenectomy; SMV, superior mesenteric vein; SpV, splenic vein

After tunneling through the dorsal aspect of the pancreas above the portal vein, pancreatic transection was performed using a linear stapler (**Fig. 3A**). However, CP was not recognized at this stage.

During dissection of the vessels supplying the uncinate process to mobilize it to the right side of the portal vein, the pancreatic parenchyma of the uncinate process was found to extend continuously from the posterior to the left side of the portal vein, fusing with the pancreatic body. At that point, a review of preoperative imaging confirmed the presence of CP (**Fig. 3B**).

However, neither preoperative imaging studies, including magnetic resonance cholangiopancreatography (MRCP), nor intraoperative findings—although intraoperative pancreatography was not performed—could clearly identify the course of the main pancreatic duct within the pancreatic head, making it impossible to classify the CP subtype according to Joseph’s classification. Based on these findings, the surgical team discussed the optimal transection plane and decided to perform an extended resection, involving further pancreatic resection beyond the dorsal–ventral pancreatic fusion site to accommodate all CP types.

With the portal vein taped and retracted ventrally, dissection was performed between the pancreas and the posterior surface of the portal vein. The dissection was 1st initiated from the right side of the portal vein and then continued from the left side, followed by separation of the fusion plane between the pancreatic parenchyma and the retroperitoneum. Approximately 1.5 cm distal to the fusion site, the pancreatic parenchyma was divided using a linear stapler (**Fig. 3C**). Subsequently, further dissection of the fusion planes between the pancreatic parenchyma and the portal vein, as well as between the pancreas and the retroperitoneum, was performed, resulting in complete mobilization of the pancreatic body from the portal vein and retroperitoneum. In this step, the multi-articulated function of the robotic system was particularly useful.

The resected pancreatic body, including the additional portion, was then mobilized to the right side of the portal vein through its posterior aspect. The fusion with the retroperitoneum was meticulously divided. Preoperative imaging studies confirmed that the right hepatic artery, which branched early from the common hepatic artery, ran dorsally to the portal vein and pancreatic parenchyma. After the extended resection of the pancreatic body, the right hepatic artery was identified during mobilization of the pancreatic head from the retroperitoneum, and careful dissection was performed to separate the pancreatic parenchyma from the artery to avoid injury. Finally, the common bile duct was transected, and the specimen was retrieved (**Fig. 3D**).

Pancreaticojejunostomy was performed using a standard duct-to-mucosa anastomosis with the modified Blumgart technique (**Supplementary Video 3**).

## DISCUSSION

CP is an asymptomatic morphological anomaly of the pancreas in which the uncinate process encircles the portal vein or superior mesenteric vein and fuses with the dorsal aspect of the pancreatic body.^1–3)^ In 1987, Sugiura et al.^11)^ reported the 1st case of CP identified intraoperatively, describing it as “a hypertrophic uncinate process of the pancreas wrapping the superior mesenteric vein and artery.” Complete fusion of the uncinate process with the pancreatic body was subsequently described by Trede.^12)^ during pancreatic resection. CP occurs in approximately 0.5%–2.5% of individuals,^1,3–5,13)^ making it a very rare variant. In pancreatic resections for CP, the incidence of postoperative pancreatic fistula is high, and determining the pancreatic transection plane according to the course of the MPD is particularly critical.^3–5)^ Therefore, identifying the MPD course preoperatively is essential, although accurate diagnosis remains challenging.^4,5)^ In a study by Harnoss et al.,^3)^ involving 17 cases of pancreatic resection, nearly half of the patients were misdiagnosed preoperatively, and CP was recognized only intraoperatively. As in our case, many instances of CP are missed on preoperative imaging and diagnosed intraoperatively.^4,5)^ Consequently, it is important to establish an appropriate surgical strategy for pancreatic resection in such cases, recognizing this clinical reality.

CP is classified based on the relationship between the fused pancreas and the splenic vein, and between the MPD and the portal or superior mesenteric vein. Karasaki et al.^1)^ classified CP as suprasplenic (type A), intrasplenic (type B), or mixed type, depending on its relationship with the splenic vein. Joseph et al.^2)^ proposed a classification into 3 types according to the MPD course. In type I, the ventral pancreatic bud fuses with the body and ductal system posterior to the portal vein. In type II, type I is associated with pancreatic divisum. In type III, only the uncinate process encases the vessels and fuses with the pancreatic body anterior to the portal vein. The most common type is type III (63%–97.5%), followed by type I (8%–22%), with type II being the rarest (0%–14.8%).^3,9)^

The optimal pancreatic transection site during pancreaticoduodenectomy in patients with CP remains debated. Pandrowala et al.^7)^ proposed different surgical strategies based on Joseph’s classification. Specifically, for type I and type II CP, extended resection—excising the entire CP to achieve a single pancreatic stump—is recommended, whereas for type III, a standard transection plane with suturing of the retroportal portion is suggested. In our case, preoperative imaging revealed no MPD dilation, and the MPD course in the pancreatic head remained unclear. Endoscopic retrograde cholangiopancreatography (ERCP),^11,14–16)^ MRCP,^7,14,17)^ and intraoperative pancreatography.^6)^ have been reported to be useful for confirming the pancreatic ductal anatomy in cases of annular pancreas. In the present case, however, detailed evaluation of the pancreatic duct by ERCP was not performed because the patient had distal bile duct cancer and the diagnosis of annular pancreas had not been established preoperatively. Moreover, as there was no dilation of the main pancreatic duct, MRCP could not clearly demonstrate the pancreatic ductal course in the pancreatic head. In addition, intraoperative pancreatography was not performed; consequently, the Joseph’s classification of CP could not be determined during surgery. Therefore, extended resection was selected as a strategy applicable to all CP types, and the final diagnosis of type II CP was confirmed histologically. No postoperative pancreatic fistula occurred, supporting extended resection as a prudent surgical choice. Previous studies have reported that extended resection may impair pancreatic endocrine or exocrine function.^1,3,6)^ However, some authors have argued that the inability to visualize a retroportal ductal structure does not necessarily exclude the presence of CP.^18)^ Accordingly, when preoperative imaging fails to clearly delineate the MPD—especially in cases with minimal MPD dilation—extended resection should be considered.

To date, 7 cases.^2,5,6,10,19)^ of pancreaticoduodenectomy for type II CP, including our case, have been reported (**Table 1**). A literature search was performed using PubMed Central (https://www.ncbi.nlm.nih.gov/pmc/) for articles published in English from May 1987 to November 2024, with the search terms “circumportal pancreas,” “periportal pancreas,” and “portal annular pancreas.” Among the 7 cases, only 2 were diagnosed preoperatively. Extended resection was performed in 6 of the 7, and none developed postoperative pancreatic fistula. Regarding the pancreatic transection line for extended resection in type II CP, Matsumoto et al.^19)^ identified the MPD fusion site using intraoperative pancreatography to determine the resection level. According to Karasaki et al.,^1)^ the average fusion length of CP was 9.4 mm. Thus, transecting the pancreas approximately 1 cm distal to the fusion site may allow the MPD to become a single duct regardless of CP type, facilitating a standard pancreatic duct-to-gastrointestinal anastomosis.^6)^

**Table 1:** Previously reported cases of type II CP undergoing pancreaticoduodenectomy

No.	Year	Author	Ref. #	Age	Gender	Karasaki’s classification	Preoperative detection of CP	Dilatation of the pancreatic duct	Variation of hepatic artery	Primary tumor	Approach	Ante-portal side of pancreas stump	Anastomosis	POPF
1	2010	Joseph	2	51	M	A	No	Yes	ND	Ampullary cancer	Open	Interrupted sutures, side to side PJ	PJ	No
2	2012	Muto	6	45	F	ND	No	No	ND	Insulinoma	Open	Extended resection	PJ	No
3	2013	Matsumoto	19	81	W	B	Yes	Yes	ND	Ampullary cancer	Open	Extended resection	PJ	No
4	2017	Luu	5	81	M	A	No	No	No	Ampullary cancer	Open	Extended resection	PJ	No
5	2017	Luu	5	60	M	A	No	Yes	Yes	Chronic pancreatitis	Open	Extended resection	PJ	No
6	2023	Parray	10	59	M	A	Yes	Yes	ND	Pancreas cancer	Open	Extended resection	ND	No
7	2025	Our case		60	M	A	No	No	Yes	Diastral common duct cancer	Robotic	Extended resection	PJ	No

CP, circumportal pancreas; ND, not described; POPF, postoperative pancreatic fistula; PJ, pancreaticojejunostomy

Two reports of RPD for CP have been published to date, and our case represents the 1st report for type II CP. Takagi et al.^8)^ described RPD for type III CP using a standard resection plane and noted that extended resection can be technically demanding in minimally invasive surgery due to its procedural complexity. They suggested that a minimally invasive approach may be more suitable for type III CP. In contrast, Imamura et al.^18)^ performed robotic distal pancreatectomy with extended resection for type IIIA CP, which was preoperatively diagnosed by imaging. They employed a planned distal-1st transection, followed by circumferential dissection of the pancreatic parenchyma from the portal vein. In our case, parenchymal transection was 1st performed above the portal vein, and CP was identified during dissection of the interface between the dorsal surface of the portal vein and the pancreatic parenchyma. This approach differed from that of Imamura et al., and may serve as a valuable reference for cases in which CP is not detected preoperatively but is instead recognized intraoperatively.

RPD for intraoperatively identified CP poses significant technical challenges even for experienced surgical teams; nevertheless, it provides unique advantages that facilitate safe and precise surgery. Given the patient’s condition and surgical tolerance, robotic surgery should be avoided when prolonged operative time is deemed inappropriate, or when dissection between the portal vein and pancreatic parenchyma is expected to be difficult and requires tactile feedback to ensure safety and curability. In such situations, conversion to open surgery should be considered. Despite these limitations, robotic assistance offers unique advantages—providing high-definition 3D visualization and precise multi-articulated instrument control that enable safe and meticulous dissection around the portal vein. These features make the robotic platform particularly useful for addressing complex pancreatic anomalies such as CP. In the present case, robotic assistance allowed the entire surgical team to share a high-resolution 3D view of the anatomical anomaly encountered intraoperatively and to discuss the surgical strategy in real time. Furthermore, the multi-articulated instruments were particularly advantageous for precise dissection between the dorsal aspect of the portal vein and the pancreatic parenchyma. Given these technical advantages, robotic surgery is expected to play an increasingly important role in treating anatomically complex pancreatic diseases in the future.

## CONCLUSIONS

CP is a rare and technically demanding anatomical variant encountered during pancreaticoduodenectomy. When the pancreatic ductal anatomy cannot be clearly identified, extended resection offers a safe and rational strategy to achieve a single-duct reconstruction and reduce postoperative complications.

Although pancreaticoduodenectomy for CP requiring extended resection has traditionally been performed via open surgery, robotic surgery, with its magnified visualization and stable instrument control, allowed a safe and minimally invasive approach even in such technically challenging cases.

This experience contributes to refining surgical strategies for CP and supports the broader application of robotic surgery in complex pancreatic procedures.

## SUPPLEMENTARY MATERIALS

Supplementary Video 1 Identification and transection of circumportal pancreas encasing the portal vein to obtain a single pancreatic duct for reconstruction.

Supplementary Video 2 Preoperative contrast-enhanced CT and 3D CT findings.

Supplementary Video 3 Intraoperative findings of RPD with extended pancreas resection.

## DECLARATIONS

### Availability of data and materials

Data will be made available on reasonable request.
